# Prevalence of Autoimmune Thyroid Disease and Associated Autoimmune Conditions in Hypothyroidism: A Cross-Sectional Study From a Tertiary Care Hospital in New Delhi

**DOI:** 10.7759/cureus.95209

**Published:** 2025-10-23

**Authors:** Shantanu Shah, Dharmander Singh, Sunil Kohli

**Affiliations:** 1 Medicine, Hamdard Institute of Medical Sciences and Research, New Delhi, IND

**Keywords:** anti-thyroid peroxidase antibodies (anti-tpo), autoimmune hypothyroidism, autoimmune thyroid disease, celiac disease and autoimmunity, subclinical hypothyroidism

## Abstract

Background: Hypothyroidism remains a significant public health issue in India, with autoimmune thyroid disease (AITD) increasingly recognized as a major contributor despite the nationwide success of iodized salt programs in addressing iodine deficiency. Identifying autoimmune associations in hypothyroidism is critical for improving diagnostic and management strategies. Objective: This study aimed to assess the prevalence of AITD and its association with other autoimmune conditions among patients with hypothyroidism. Methods: A cross-sectional study was conducted at the Department of Medicine, Hakeem Abdul Hameed Centenary Hospital, New Delhi, between June 2023 and May 2024. A total of 165 adult patients (≥18 years) with overt or subclinical hypothyroidism were enrolled. Clinical history, demographic data, anthropometric measurements, and biochemical investigations were obtained. Thyroid profiles, anti-thyroid peroxidase (anti-TPO) antibodies, and anti-tissue transglutaminase (anti-TTG) antibodies were assessed. Data were analyzed using SPSS v21.0. Statistical significance was defined at p<0.05. Results: Of 165 participants, 124 (75.2%) were female and 41 (24.8%) were male, with a female-to-male ratio of approximately 3:1. The predominant age group was 41-60 years (41.2%). Elevated anti-TPO antibodies were detected in 130 patients (78.8%), confirming AITD as the leading etiology. A statistically significant correlation was observed between autoimmune disorders and anti-TPO positivity (p<0.001). Additionally, 15.4% of subjects were anti-TTG positive, all of whom also exhibited elevated anti-TPO antibodies (p=0.008). Conclusion: AITD accounted for the majority of hypothyroidism cases in this study, with a significant association observed between hypothyroidism and other autoimmune conditions, including celiac disease. These findings underscore the importance of screening hypothyroid patients for autoimmunity and highlight the need for prospective studies to establish causal pathways and long-term outcomes.

## Introduction

Thyroid disorders represent a major health issue in India, with an estimated 42 million individuals affected [1]. Hypothyroidism, which is the most common thyroid disorder, particularly impacts women as well as older adults. Approximately 10%-12% of women in India are affected by hypothyroidism, with a higher incidence observed in individuals over the age of 35 [2]. The prevalence of hypothyroidism in India is estimated to be around 10.95%. While iodine deficiency was historically the predominant cause, the introduction of iodized salt has largely addressed this issue. Recently, there has been a trend to use pink salt (Himalayan salt), but exclusive use of pink (Himalayan) salt may worsen or precipitate hypothyroidism due to its low and inconsistent iodine content, but at the same time, no studies have been done to show this evidence. Hence, iodized salt remains essential for maintaining adequate iodine intake and normal thyroid hormone synthesis. Hypothyroidism remains prevalent even after adequate iodine supplementation, as evidenced by the rise in self-reported thyroid disorders, from 2.2% in the National Family Health Survey IV (NFHS-IV, 2015-2016) to 2.9% in NFHS-V (2019-2021), a trend increasingly driven by autoimmune etiologies such as Hashimoto’s thyroiditis. A recent systematic review estimated the global prevalence of Hashimoto’s thyroiditis at 7.5% (95% CI: 5.7-9.6%), with higher rates in low-to middle-income regions [3]. This study investigates the prevalence of autoimmune hypothyroid disease, characterized by elevated anti-thyroid peroxidase (anti-TPO) antibodies, and its association with other autoimmune conditions, particularly celiac disease, in patients with hypothyroidism.

## Materials and methods

Study design and setting

This was a cross-sectional, observational study conducted in the Department of Medicine at Hakeem Abdul Hameed Centenary Hospital, Hamdard Institute of Medical Sciences and Research, New Delhi, between June 2023 and May 2024. The hospital is a tertiary care teaching institution catering to a large urban and semi-urban population, providing an appropriate setting to study the prevalence and autoimmune associations of hypothyroidism.

Study population

A total of 165 adult patients (aged ≥18 years) who were diagnosed with either overt or subclinical hypothyroidism were enrolled after providing informed consent. Patients who were not willing to participate were excluded from the study.

Definitions

Overt hypothyroidism was defined as a TSH value >10 μIU/mL with reduced serum free T3 (<1.88 pg/mL) and/or free T4 (<0.70 ng/dL). Subclinical hypothyroidism was defined as an elevated TSH (>4.94 μIU/mL) with normal levels of free T3 and free T4.

Clinical and laboratory assessment

All participants underwent clinical evaluation, including demographic characteristics (age and sex), and a relevant history of thyroid or other autoimmune conditions. Blood samples were collected after overnight fasting for thyroid function tests. Thyroid function tests, including TSH, free T3, and free T4, were measured using the ARCHITECT i2000SR analyzer (Abbott Laboratories, Chicago, IL, USA). Anti-TPO antibodies were tested using a standardized enzyme-linked immunosorbent assay (ELISA), and anti-tissue transglutaminase (anti-TTG) antibodies were measured to screen for celiac disease in subjects with associated symptoms.

Ethical considerations

The study was approved by the Institutional Ethics Committee of Hamdard Institute of Medical Sciences and Research. Written informed consent was obtained from all participants prior to inclusion.

Statistical analysis

Data was compiled in Microsoft Excel (Microsoft Corp., Redmond, WA, USA) and analyzed using SPSS version 21.0 (IBM Corp., Chicago, USA). Descriptive statistics were calculated for demographic and biochemical parameters. Continuous variables were expressed as mean ± standard deviation (SD), and categorical variables as frequencies and percentages. Group comparisons were performed using Student’s t-test for continuous variables and chi-square test or Fisher’s exact test for categorical variables, as appropriate. Statistical significance was defined as a p-value <0.05.

## Results

A total of 165 subjects with hypothyroidism, either overt or subclinical, were enrolled in this study. Of these, 124 (75.2%) were female and 41 (24.8%) were male, yielding a female-to-male ratio of approximately 3:1 (Table 1).

**Table 1:** Gender distribution of study population Data are presented as N (numerical value) and % (percentage). The distribution of study participants (n=165) by gender showed that females constituted a significantly larger proportion compared to males.

Gender	N	%
Male	41	24.8
Female	124	75.2
Total	165	100.0

The age distribution showed that the largest proportion of study subjects fell within the 41-60 year age group (41.2%), followed by those aged >60 years (30.3%) and lastly, 18-40 years (28.5%) (Table 2).

**Table 2:** Age distribution of study population Data are presented as N (numerical value) and % (percentage). Age distribution of hypothyroid patients showed that the highest proportion belonged to the 41-60 years age group, followed by those aged over 60 years.

Age group (years)	N	%
18-40	47	28.5
41-60	68	41.2
>60	50	30.3
Total	165	100.0

With respect to autoimmune status, elevated anti-TPO antibodies were detected in 130 of the study subjects (78.8%), while 35 subjects (21.2%) had normal anti-TPO levels (Table 3). The prevalence of anti-TPO positivity was significantly higher among females compared to males (Table 4).

**Table 3:** Anti-TPO antibody status Data are presented as N (numerical value) and % (percentage). The prevalence of elevated anti-TPO antibodies among the study subjects was 78.8%, confirming AITD as the predominant etiology. anti-TPO: anti-thyroid peroxidase; AITD: autoimmune thyroid disease

Anti-TPO	N	%
Elevated	130	78.8
Normal	35	21.2
Total	165	100.0

**Table 4:** Gender-wise distribution of anti-TPO status ^*^P<0.05 considered statistically significant. Data are presented as N (numerical value) and % (percentage). Percentages are calculated within each gender group. Anti-TPO indicates anti-TPO antibody. Statistical test: χ²=5.05, df=1, p=0.0001 (p<0.001; statistically significant). A significant association was observed between gender and anti-TPO antibody elevation, indicating a higher prevalence among females. anti-TPO: anti-thyroid peroxidase; χ²: Chi-square; df: degrees of freedom; p: probability value

Gender	Anti-TPO elevated, n (%)	Anti-TPO normal, n (%)	Total, n	χ² (df)	P-value
Male	23 (56.1)	18 (43.9)	41		
Female	107 (86.3)	17 (13.7)	124		
Total	130 (78.8)	35 (21.2)	165	15.05 (1)	0.0001^*^

Analysis of associated autoimmune conditions demonstrated that 36 subjects (27.7%) with elevated anti-TPO antibodies also had another autoimmune disorder, whereas none of the subjects with normal anti-TPO levels had an associated autoimmune disease. This association was statistically significant (χ² test, p<0.001). Furthermore, anti-TTG positivity was observed in 20 subjects (15.4%), all of whom were anti-TPO positive. The relationship between anti-TTG positivity and anti-TPO elevation was also statistically significant (Fisher’s exact test, p=0.008) (Table 5).

**Table 5:** Association of autoimmune conditions with anti-TPO positivity Data are presented as N (numerical value) and % (percentage). The association between autoimmune conditions (including celiac disease, as indicated by anti-TTG positivity) and anti-TPO antibody status was assessed. Autoimmune disorders were significantly more prevalent in anti-TPO-positive patients (χ²=10.83, p<0.001). Anti-TTG positivity was also significantly associated with anti-TPO elevation (Fisher’s exact test, OR=∞, p=0.008). Statistical analysis was performed using the Chi-square test; p<0.05 was considered statistically significant. anti-TPO: anti-thyroid peroxidase; anti-TTG: anti-tissue transglutaminase

Condition	Anti-TPO elevated (N, %)	Anti-TPO normal (N, %)	P-value
Autoimmune disease (Yes)	36 (27.7%)	0 (0.0%)	<0.001
Autoimmune disease (No)	94 (72.3%)	35 (100.0%)	
Anti-TTG positive	20 (15.4%)	0 (0.0%)	0.008
Anti-TTG negative	110 (84.6%)	35 (100.0%)	

In summary, the majority of hypothyroid subjects demonstrated evidence of autoimmunity, with anti-TPO positivity strongly associated with both female gender and the presence of additional autoimmune conditions, particularly celiac disease.

## Discussion

This study demonstrates that AITD, as reflected by elevated anti-TPO antibody levels in the study subjects, is the predominant etiology of hypothyroidism. The prevalence of anti-TPO positivity (78.8%) observed here is notably higher than in some earlier Indian studies, suggesting a rising trend of autoimmune hypothyroidism in the Indian population. For example, Sharma et al. reported anti-TPO positivity in approximately 58.3% of subjects in their study with subclinical hypothyroidism, which is significantly lower than the rate we found in both subclinical and overt hypothyroidism [4]. Similarly, other Indian data also indicate an increasing recognition of autoimmunity as the leading cause of hypothyroidism in iodine-sufficient regions [2].

The female predominance in our study (75.2%) aligns with the well-documented gender disparity in AITD. Several epidemiological studies have shown that women are almost three times more likely to develop hypothyroidism, with the risk increasing further in middle age and beyond [3]. Our study findings confirm this trend and emphasize the role of associated sex hormones and genetic predisposition in driving autoimmunity.

An important observation from this study is the statistically significant association between anti-TPO positivity and other autoimmune diseases. In particular, all subjects with anti-TTG positivity were also anti-TPO positive, highlighting a strong correlation between autoimmune hypothyroidism and celiac disease. This finding is supported by global data. Antonelli et al. reported that autoimmune thyroiditis frequently clusters with other autoimmune conditions such as celiac disease, type 1 diabetes mellitus, and pernicious anemia, reflecting shared genetic and immunological pathways [5]. Similarly, Meloni et al. demonstrated a higher prevalence of celiac disease in adults as well as children with autoimmune thyroiditis when compared to the general population [6]. These associations highlight the need for a multidisciplinary approach in evaluating overt or subclinical hypothyroid patients, as early detection of coexisting autoimmune conditions, especially celiac disease, may improve long-term outcomes.

Our findings also resonate with the landmark Whickham Survey, which identified thyroid autoimmunity as the most important risk factor for the development of hypothyroidism, particularly in women and the elderly [7]. More recent systematic reviews and meta-analyses also confirm that Hashimoto’s thyroiditis is now the most common cause of hypothyroidism in iodine-sufficient populations, with prevalence rates varying by geography but consistently showing higher rates in low as well as middle-income countries [3].

From a clinical perspective, our results underscore two important implications. First, anti-TPO antibody testing should be routinely considered in the evaluation of hypothyroid patients, as it not only establishes the autoimmune etiology but also identifies patients who may be at risk of developing other autoimmune conditions. Second, given the significant association between anti-TPO and anti-TTG positivity, screening for celiac disease should be considered in hypothyroid patients, particularly those presenting with gastrointestinal symptoms, anemia, or unexplained weight loss.

In conclusion, this study reinforces the changing epidemiology of hypothyroidism in India, where autoimmune thyroiditis has overtaken iodine deficiency as the predominant cause. The significant association with other autoimmune conditions, particularly celiac disease, highlights the systemic nature of autoimmunity and the need for broader screening and integrated care.

This study has certain limitations. First, being a single-center, cross-sectional study with a modest sample size, the findings may not be generalizable. Second, while anti-TTG antibody testing was used to identify possible celiac disease in symptomatic patients, confirmatory diagnosis with endoscopic biopsy, the gold standard, could not be performed in all cases, and asymptomatic individuals were not screened. Therefore, the observed association between hypothyroidism and celiac disease should be interpreted with caution. Despite these limitations, our study provides valuable baseline data on the autoimmune profile of hypothyroid patients in an Indian tertiary care setting and highlights important associations that warrant larger, multicenter, and prospective studies for confirmation. This work can serve as a foundation for future research exploring broader autoimmune screening in hypothyroid patients.

## Conclusions

This study demonstrates that AITD is the predominant cause of hypothyroidism in our study subjects, with nearly 79% of patients showing anti-TPO antibody positivity. The findings also confirmed a strong female preponderance, almost three times more common, with middle-aged adults being most frequently affected, which aligns with the known demographic distribution of autoimmune hypothyroidism. Importantly, a statistically significant association was observed between hypothyroidism and other autoimmune conditions, particularly celiac disease, as all anti-TTG-positive patients were also anti-TPO positive. These results highlight that autoimmunity has overtaken iodine deficiency as the leading etiology of hypothyroidism in India and emphasize the clinical utility of anti-TPO antibody testing not only for establishing a diagnosis but also for identifying patients who may be at an increased risk for coexisting autoimmune conditions. Although this study was limited by its single-center design and the lack of confirmatory endoscopic biopsy for celiac disease in all subjects who were anti-TTG positive, it provides valuable baseline evidence and serves as a foundation for future multicenter, longitudinal research.
